# Supplementary material for: The effects of upper body blood flow restriction training on muscles located proximal to the applied occlusive pressure: A systematic review with meta-analysis
Source: PLoS One. 2023 Mar 23;18(3):e0283309. doi: 10.1371/journal.pone.0283309 (PMC10035935; doi:10.1371/journal.pone.0283309)
Supplement: S1 File — (PDF) [file pone.0283309.s001.pdf]

Supplementary table 1: Search strategy and results in databases

Key words	Results in Medline (Pubmed)	Results in CINAHL	Results in SportDiscus	Results in Scopus
S1: (BFR[Title/Abstract]) OR (blood flow restriction [Title/Abstract]) OR (kaatsu training [Title/Abstract]) OR (occlusion training [Title/Abstract]) OR (vascular occlusion training [Title/Abstract])	2,245	353	511	482
S2: (arm [Title/Abstract]) OR (shoulder [Title/Abstract]) OR (upper limb [Title/Abstract]) OR (upper extremity [Title/Abstract]) OR (rotator cuff [Title/Abstract])	275,234	20,779	12,040	159
S1 AND S2	100	96	122	12

Supplementary table 2. Measurement method of the outcome of interest in strength, muscle size, and tendon thickness

Study	Comparators	Outcome for strength	Testing specification
Bowman et al.,2020	LL-BFRT vs LL-RT	Internal rotation (isokinetic)	No information with regards to the testing position.
Bowman et al.,2020	LL-BFRT vs LL-RT	External rotation (isokinetic)	Isokinetic internal rotation and external rotation measurements were performed at 180°/s, 270/s, and 300°/s using the Biodex System 3 (Biodex Medical Systems, Shirley, NY, USA).
Bowman et al.,2020	LL-BFRT vs LL-RT	Abduction (MVIC)	No information for the testing position
Bowman et al.,2020	LL-BFRT vs LL-RT	Scaption (MVIC)	No information for the testing position
Bowman et al.,2020	LL-BFRT vs LL-RT	Flexion (MVIC)	No information for the testing position
Lambert et al.,2021	LL-BFRT vs LL-RT	Internal rotation 90º (MVIC)	Prone internal rotation at 90° of shoulder abduction
Lambert et al.,2021	LL-BFRT vs LL-RT	External rotation 90° (MVIC)	Prone external rotation at 90° of shoulder abduction
Lambert et al.,2021	LL-BFRT vs LL-RT	Internal rotation 0º (MVIC)	Seated internal rotation at 0° of shoulder abduction
Lambert et al.,2021	LL-BFRT vs LL-RT	External rotation 0º (MVIC)	Seated external rotation at 0° of shoulder abduction
Lambert et al.,2021	LL-BFRT vs LL-RT	Scaption (MVIC)	Seated scaption at 90º of relative flexion.
Lambert et al.,2021	LL-BFRT vs LL-RT	Flexion (MVIC)	Seated forward flexion at 90º of shoulder abduction

Brumit et al.,2020	LL-BFRT vs LL-RT	Abduction (MVIC) - supraspinatus	Subject assumes a sitting position on the table. The shoulder is in a neutral position and abducted to 30°. The elbow is flexed to 90°. The therapist applies resistance laterally to the upper extremity above the elbow region.
Brumit et al.,2020	LL-BFRT vs LL-RT	External rotation 90° (MVIC)	Prone external rotation at 90° of shoulder abduction
Yamanaka et al., 2012	LL-BFRT vs LL-RT	Bench press (1RM)	On a bench. The 1RM was determined when the participant successfully completed an exercise with the heaviest barbell throughout the full range of motion
Yasuda et al., 2010	LL-BFRT vs LL-RT	Bench press (1RM)	Maximal dynamic strength (1-RM) was assessed using a free-weight flat bench press. A test was considered valid only when the subject used proper form and completed the entire lift in a controlled manner without assistance
Green et al.,2020	LL-BFRT vs HL-RT	Pectoralis major (MVIC)	No description of the measurement – measurement methodology illustrated in figure 1: Supine lying with arm at 90° of horizontal abduction
Green et al.,2020	LL-BFRT vs HL-RT	Lower trapezius (MVIC)	No description of the measurement – measurement methodology illustrated in figure 1: Prone lying with arm at 180° of horizontal abduction. Resistance in horizontal extension of the proximal arm
Green et al.,2020	LL-BFRT vs HL-RT	External Rotation (MVIC)	No description of the measurement – measurement methodology illustrated in figure 1: Prone external rotation at 90^0 of shoulder abduction
Green et al.,2020	LL-BFRT vs HL-RT	Serratus anterior (MVIC)	No description of the measurement – measurement methodology illustrated in figure 1: Upper arm in 90° of shoulder flexion and reach
Green et al.,2020	LL-BFRT vs HL-RT	Rhomboids (MVIC)	No description of the measurement – measurement methodology illustrated in figure 1: Prone with hand behind the back and resistance was applied on the lateral boarder of the scapula in scapular retraction movement
Thiebaut et al.,2013	LL-BFRT vs HL-RT	Seated row (1RM)	1RM was calculated by following standard previously described procedures (Baechle et al., 2000)
Thiebaut et al.,2013	LL-BFRT vs HL-RT	Chest press (1RM)	1RM was calculated by following standard previously described procedures (Baechle et al., 2000)
Thiebaut et al.,2013	LL-BFRT vs HL-RT	Shoulder press (1RM)	1RM was calculated by following standard previously described procedures (Baechle et al., 2000)
Salyers,2017	LL-BFRT vs HL-RT	Bench press (1RM)	For completion of the bench press, subjects laid supine on the bench. Five points of body contact were maintained during the entire lift: (a) head, (b) shoulders, (c) buttocks, (d) right foot, and (e) left foot. The bar was then lifted off the rack by the participant, with the assistance from the spotter if needed and lowered to the chest and lifted off their chest until full elbow extension was achieved
Yasuda et al.,2011	LL-BFRT vs HL-RT	Bench press (1RM)	Maximal dynamic strength (1-RM) was assessed using a free-weight flat bench press. A test was considered valid only when the subject used proper form and completed the entire lift in a controlled manner without assistance

Yasuda et al.,2011	LL-BFRT vs No exercise	Bench press (1RM)	Maximal dynamic strength (1-RM) was assessed using a free-weight flat bench press. A test was considered valid only when the subject used proper form and completed the entire lift in a controlled manner without assistance
**Study**	**Comparators**	**Outcome for muscle size**	**Measurement method**
Yasuda et al.,2011	LL-BFRT vs No exercise	Pectoralis major via MRI (cm^2)	Subjects rested quietly in the magnet bore in a supine position, with their arms extended along their trunk. Continuous transverse images with 10-mm slice thickness were obtained from the upper right side of the body, including the arm. Triceps brachii and pectoralis major muscle CSAs of three contiguous slices for muscle belly were averaged together for statistical analysis.
Yasuda et al.,2010	LL-BFRT vs LL-RT	Pectoralis major via US (cm)	Muscle size was measured using B-mode ultrasound (Aloka SSD-500, Tokyo, Japan) at two anatomical sites [chest (at the site between third and fourth of costa under the clavicle midpoint) and posterior upper arm (at 60% distal between the lateral epicondyle of the humerus and the acromial process of the scapula)] of the left side as has been described previously (Abe et al., 1994, 2000). Briefly, the measurements were carried out while the subjects stood with their elbows extended and relaxed. A 5-MHz scanning head was placed on the measurement site without depressing the dermal surface. The subcutaneous adipose tissue–muscle interface and the muscle–bone interface were identified from the ultrasonic image, and the distance between two interfaces was taken as muscle thickness.
Yamanaka et al., 2012	LL-BFRT vs LL-RT	Upper chest girth (cm)	Upper chest girth was measured around the upper latissimus dorsi and below the armpits at the end of normal expiration.
Yamanaka et al., 2012	LL-BFRT vs LL-RT	Lower chest girth (cm)	Lower chest girth was measured at nipple level at the end of normal expiration.
Lambert et al.,2021	LL-BFRT vs LL-RT	Shoulder lean mass via DEXA (g)	For shoulder region analysis, the region of interest parameters were templated to individual participants based on skeletal landmarks in their initial scan that were then subsequently used for the post-training measure. The 2D landmarks included the cervical vertebrae traced to the top of the first rib, down the outer edge of the rib cage to the location at which the scapula visually intersected the ribs, across the humerus (parallel to the bottom of the scan), and then around the upper arm, shoulder, and trapezius muscles, ending at the highest cervical vertebra below the jawbone.
Salyers,2017	LL-BFRT vs HL-RT	Chest girth via tape (cm)	A measure at the nipple level, under the arms

Yasuda et al.,2011	LL-BFRT vs HL-RT	Pectoralis major via MRI (cm^2)	Subjects rested quietly in the magnet bore in a supine position, with their arms extended along their trunk. Continuous transverse images with 10-mm slice thickness were obtained from the upper right side of the body, including the arm. Triceps brachii and pectoralis major muscle CSAs of three contiguous slices for muscle belly were averaged together for statistical analysis.
Thiebaut et al.,2013	LL-BFRT vs HL-RT	Pectoralis major via US (cm)	Muscle thickness (MTH) was measured by B-mode ultrasound (Aloka SSD-500, Tokyo) the week prior to and the week after 8 weeks of training by a single experienced technician. A 5-MHz scanning head was placed on the skin without depressing the dermal surface. Distortion of tissue due to excess compression was eliminated by observing that no movement of tissue occurred in the real time ultrasound image. Pectoralis major MTH was also measured at the clavicular midpoint and between the third and fourth costa (Yasuda et al., 2010), and deltoid MTH was measured on the lateral surface of the shoulder and at the thickest MTH of the deltoid. Muscle thickness was determined as the distance between the adipose tissue–muscle interface and muscle–bone interface from the ultrasound image.
Thiebaut et al.,2013	LL-BFRT vs HL-RT	Deltoid via US (cm)	
Thiebaut et al.,2013	LL-BFRT vs HL-RT	Trunk bone-free mass via DEXA (Kg)	Dual energy X-ray absorptiometry (DXA, GE Medical Systems, Lunar Prodigy, encore 2010 software version 13.31.016) was used to assess total bone-free lean body mass using a total body scan in all participants the week prior to training and the week after training.
**Study**	**Comparators**	**Outcome for tendon thickness**	**Measurement method**
Brumit et al.,2020	LL-BFRT vs LL-RT	Supraspinatus tendon thickness (US mm)	Long- and short-axis views of the supraspinatus were obtained. The footprint of supraspinatus was identified in long axis at the superior facet of the great tuberosity. The transducer was turned 90° to obtain a short axis image of the supraspinatus bringing into view the long head of biceps tendon as well. The thickness of the supraspinatus tendon was measured in the short axis at 3 points (10, 20, and 30 mm) lateral to the long head of the biceps tendon.30 The average of these 3 points was used to represent the thickness of the tendon.
Abbreviations: BFRT, blood flow restriction training; CSA, cross sectional area; DEXA, dual energy X-ray absorptiometry; HI-RT, high intensity resistance training; LL-RT, low load resistance training; MRI, magnetic resonance imaging; MTH, muscle thickness; MVIC, maximal voluntary isomeric contraction; RM, repetition maximum; US, ultrasound;			

Supplementary figure 1. Forest plots depicting studies using LL-BFRT compared to studies using LL-RT in muscle strength presenting significant statistical heterogeneity (I^2>75%)

a

	Experimental			Control				Std. Mean Difference	
**Study or Subgroup**	**Mean**	**SD**	**Total**	**Mean**	**SD**	**Total**	**Weight**	**IV, Random, 95% CI**	**Year**
Lambert et al.,2021	16.1	0.7	16	14.7	0.7	16	48.6%	1.95 [1.09, 2.81]	
Brumitt et al.,2020	13.54	4.94	24	13.35	5.7	22	51.4%	0.04 [-0.54, 0.61]	
**Total (95% CI)**			**40**			**38**	**100.0%**	**0.96 [-0.91, 2.84]**	

Heterogeneity: Tau² = 1.69; Chi² = 13.10, df = 1 (P = 0.0003); I² = 92%
Test for overall effect: Z = 1.01 (P = 0.31)

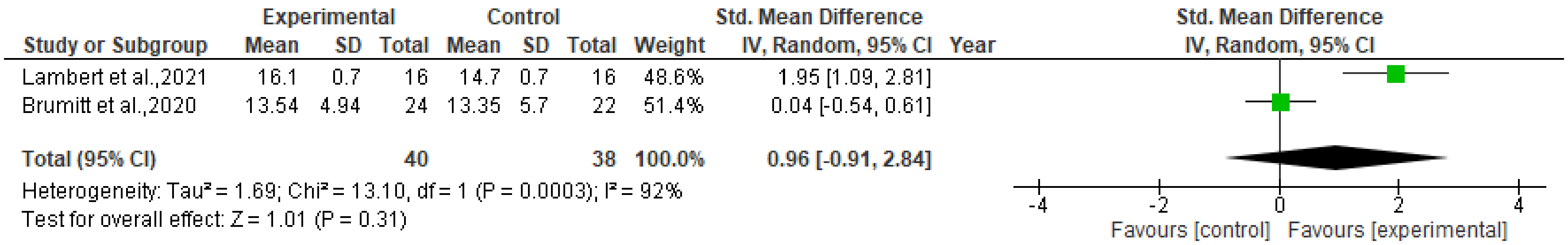

b

	Experimental			Control				Std. Mean Difference	
**Study or Subgroup**	**Mean**	**SD**	**Total**	**Mean**	**SD**	**Total**	**Weight**	**IV, Random, 95% CI**	**Year**
Bowman et al.,2020	9.04	4.49	14	4.27	3.33	10	48.6%	1.14 [0.25, 2.02]	
Lambert et al.,2021	12.4	0.2	16	12.5	0.2	16	51.4%	-0.49 [-1.19, 0.22]	
**Total (95% CI)**			**30**			**26**	**100.0%**	**0.30 [-1.29, 1.89]**	

Heterogeneity: Tau² = 1.15; Chi² = 7.91, df = 1 (P = 0.005); I² = 87%
Test for overall effect: Z = 0.37 (P = 0.71)

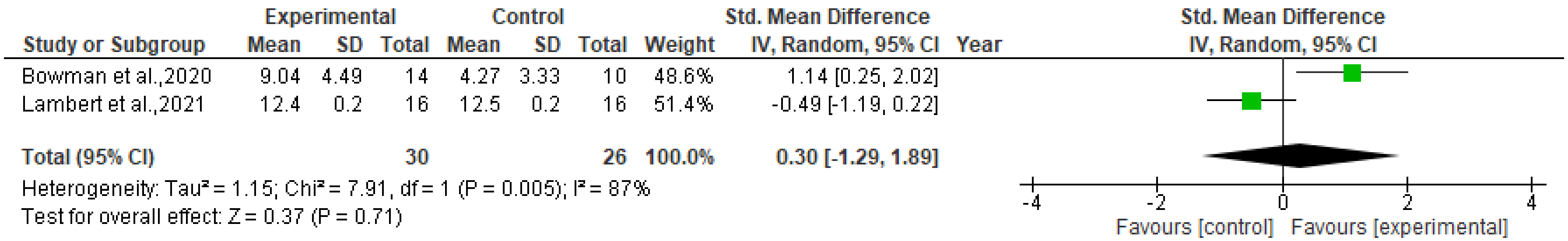

c

Study or Subgroup	Experimental Mean	Experimental SD	Experimental Total	Control Mean	Control SD	Control Total	Weight	Std. Mean Difference IV, Random, 95% CI	Year
Bowman et al.,2020	8.56	4.1	14	4.71	3.14	10	45.5%	0.99 [0.13, 1.86]	
Brumitt et al.,2020	18.86	3.46	24	19.27	4.1	22	54.5%	-0.11 [-0.69, 0.47]	
**Total (95% CI)**			**38**			**32**	**100.0%**	**0.39 [-0.68, 1.47]**	

Heterogeneity: Tau² = 0.46; Chi² = 4.28, df = 1 (P = 0.04); I² = 77%

Test for overall effect: Z = 0.72 (P = 0.47)

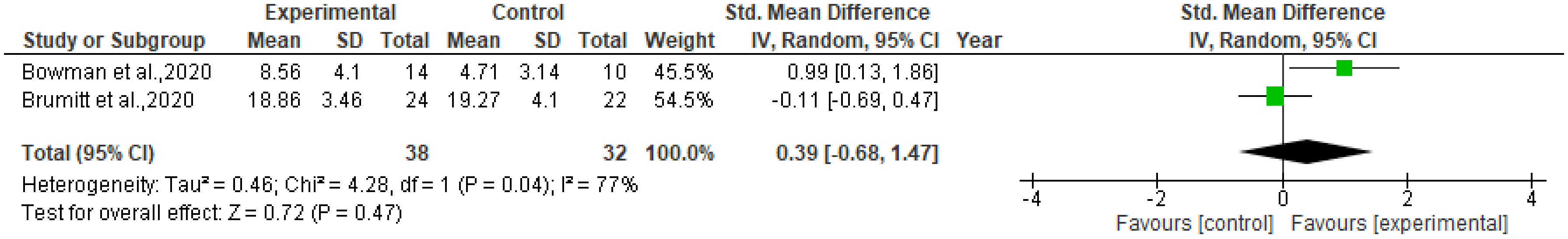

Forest plots comparing low-load resistance training with blood flow restriction (LL-BFR) and low-load resistance training alone (LL-RT) on muscle strength. a) Prone shoulder external rotation (dynamometry in kgs), b) Shoulder scaption (dynamometry in kg), and c) Shoulder abduction (dynamometry in kgs).

Abbreviations: CI, confidence interval; IV, inverse variance; Random, random effects model; SE, standard error.
