# Supplementary material for: The effects of upper body blood flow restriction training on muscles located proximal to the applied occlusive pressure: A systematic review with meta-analysis
Source: PLoS One. 2023 Mar 23;18(3):e0283309. doi: 10.1371/journal.pone.0283309 (PMC10035935; doi:10.1371/journal.pone.0283309)
Supplement: Attachment — Submitted filename: Comments to the authors.docx [file pone.0283309.s003.docx]

**Comments to the author(s)**

The authors included relevant articles after a comprehensive literature search (including manual search through the references from articles and abstracts) and provided a valuable report. Inclusion and exclusion criteria as well as number of evaluated studies are clearly stated. However, the number of included studies and the heterogeneity of the study characteristics make the results less generalizable (participant activity level [trained (7), untrained (2)], number of sessions per week [2-6 sessions per week], duration of the intervention [ 2-8 weeks], varying exercise loads [additionally in some studies the control group performed relatively higher intensity exercises (references: 35, 36, 39, 40)] as well as change in exercise load throughout the intervention program are quite heterogeneous across studies).

The paper was submitted to the journal as “Research Article”. However, the journal PLOS ONE does not consider systematic review and meta-analysis in this category. Here’s the journal statement on the subject: “Research articles must report on original research that contributes to the base of academic knowledge. Reviews, essays, opinion pieces, hypothesis papers, and other items of secondary literature are not considered.”

Abstract

-Line #18- Reformulate the sentence. For example, “Blood flow restriction combined with low load resistance training (LL-BFRT)”.

-Line #22- Change the abbreviation as “(LL-RT, HL-RT)”.

Introduction

-Line #45- It is suggested to remove the word “minimum”. The authors may type “~60-70%”.

-Line #50- The sentence may need to be reformulated. For example, “Recently, significant attention has been drawn to low load resistance training (LL-RT) combined with blood flow restriction (BFR), which involves a parallel partial restriction of the arterial flow and complete occlusion of the venous return of the exercised limb (3, 4).”

-Line #56- This paragraph is quite informative.

-Line #67- It may be more useful if the authors provide underlying reasons for these possible mechanisms.

-Line #70- The authors need to remove the word “apparent”.

-Line #73- The authors need to explain why “the large muscle groups of the upper extremity” is a possible limitation for the application of BFRT, especially compared to lower body.

Line #75- This last sentence is not a great match for the paragraph. The first two sentences in the paragraph addresses the understudied muscle groups of the upper extremity and provides possible explanations for it. The last sentence, on the other hand, is not related to these.

Line #82- Edit as “such as changes in muscle size and strength”.

Methods

-Line #93- The study objective states that “the main objective of this systematic review of randomized controlled trials (RCTs) was to evaluate in healthy individuals the effectiveness of LL-BFRT...”. However, the population for eligibility is different here in line #93. Please reformulate.

-Line #136- The authors are suggested to reformulate this sentence. Particularly, the beginning of it (“As measures of intervention effect, given that the outcomes were continuous, ...”) is not very clear.

-Line #152- The authors need to provide references for the categorization of training loads. These subgroups (> 60% 1RM for HL; >40 1RM for LL) do not agree with some of the studies referenced in the Introduction section and are relative.

-Line #154- The authors may need to explain why they are including studies with a non-exercising control group. Please also examine the study objective (at the end of the Introduction section) accordingly. This line also contradicts with the statement in line #191 (“The control group used the same exercises with the BFRT group in every study..”).

Results

-Line #181- In Table 2, third row (Brumitt et al., 2020), please indicate if the significant difference is compared to baseline levels (within-group).

-Line #181- In Table 2, 8th row (Yamanak et al., 2012), a comma (,) proceeds the p (p,0.05). Please reformulate.

-Line #181- In Table 2, 9th row (Yasuda et al., 2010) “physiological adaptations and main findings” is not clear, please reformulate.

-Line #190- Understandably the authors used the term “fatigue”, since the referenced article also used that term. However, it could be more accurate to use a term such as “volitional exhaustion” or “until participants' expressed inability to continue with the task”.

-Line #223- The referenced study (39) included 40 participants in total and had 4 groups. It could be better if the authors reformulated this sentence similar to this one: “...one study of moderate quality and high risk of bias (39) showed no difference in bench press strength between LL-BFRT (n=10) and no exercise (n=10)...”.

-Line #259- Add space between “population” and “(40)”. Please also check the whole paper for similar edits (for example line #334).

Discussion

-Line #295- The authors summarized the main findings clearly in this paragraph.

-Line #312- The authors cited the reference #44 (Centner et al., 2019), however it is not related to the statement made in that sentence. Centner et al., (2019) did not include a LL-RT group, rather they had a HL-RT group.

-Line #317- This sentence is not clear. The authors need to explain what they mean by “fatiguing effect of synergistic muscles”.

-Line #390-422- The authors stated the study limitations and conclusions clearly. The authors may include that the number of studies reviewed did not allow them to perform some of the standard analyses.

-The authors seem to have cited related and up to date references.
