# Supplementary material for: The effects of upper body blood flow restriction training on muscles located proximal to the applied occlusive pressure: A systematic review with meta-analysis
Source: PLoS One. 2023 Mar 23;18(3):e0283309. doi: 10.1371/journal.pone.0283309 (PMC10035935; doi:10.1371/journal.pone.0283309)
Supplement: Attachment — Submitted filename: Response to Reviewers.docx [file pone.0283309.s004.docx]

| **Reviewer #1: Comments to the Author** | **Authors’ responses** | **Changes in text** |
| --- | --- | --- |
| The authors included relevant articles after a comprehensive literature search (including manual search through the references from articles and abstracts) and provided a valuable report. Inclusion and exclusion criteria as well as number of evaluated studies are clearly stated. However, the number of included studies and the heterogeneity of the study characteristics make the results less generalizable (participant activity level [trained (7), untrained (2)], number of sessions per week [2-6 sessions per week], duration of the intervention [2-8 weeks], varying exercise loads [additionally in some studies the control group performed relatively higher intensity exercises (references: 35, 36, 39, 40)] as well as change in exercise load throughout the intervention program are quite heterogeneous across studies). | We thank the reviewer for the constructive comments and feedback. | No changes |
| The paper was submitted to the journal as “Research Article”. However, the journal PLOS ONE does not consider systematic review and meta-analysis in this category. Here’s the journal statement on the subject: “Research articles must report on original research that contributes to the base of academic knowledge. Reviews, essays, opinion pieces, hypothesis papers, and other items of secondary literature are not considered.” | We apologize for this mistake. We will submit the revision of the manuscript under the correct category. | No changes |
| The manuscript presented in an intelligible fashion and written in standard English. | We thank the reviewer for the feedback. | No changes |
| Abstract  -Line #18- Reformulate the sentence. For example, “Blood flow restriction combined with low load resistance training (LL-BFRT)”. | Thank you for your comment. Sentence has been reformulated as you suggested. | *“Blood flow restriction combined with low load resistance training (LL-BFRT)….”* |
| -Line #22- Change the abbreviation as “(LL-RT, HL-RT)”. | The abbreviation has been changed. | *“….low load-, or high load resistance training (LL-RT, HL-RT) on musculature…”* |
| Introduction  -Line #45- It is suggested to remove the word “minimum”. The authors may type “~60-70%”. | The word has been removed and the percentages were modified accordingly. | *“According to the American College of Exercise Medicine a resistance training load of ~60-70% of the one repetition maximum (1RM)….”* |
| -Line #50- The sentence may need to be reformulated. For example, “Recently, significant attention has been drawn to low load resistance training (LL-RT) combined with blood flow restriction (BFR), which involves a parallel partial restriction of the arterial flow and complete occlusion of the venous return of the exercised limb (3, 4).” | We thank the reviewer for the suggestion. Changes have been made. | *“Recently, significant attention has been drawn to low load resistance training (LL-RT) combined with blood flow restriction (BFR), which involves a parallel partial restriction of the arterial flow and complete occlusion of the venous return of the exercised limb (3, 4).”* |
| -Line #56- This paragraph is quite informative. | We thank the reviewer for the feedback. | No changes. |
| -Line #67- It may be more useful if the authors provide underlying reasons for these possible mechanisms. | We thank the reviewer for the suggestion. Given that several primary mechanisms have been proposed such as: mechanical tension, metabolic stress, and mechanotransduction; as well as secondary mechanisms such as: muscle damage, anabolic hormonal response due to anabolic stress, intracellular hydration and cell swelling, acute production of reactive oxygen species (including nitric oxide),  transcription of highly conserved stress proteins called heat shock proteins, and increased recruitment of type II muscle fibres – we decided not to edit the section due to the necessary length and complexity of this addition which we feel would distract from the primary message. | No changes. |
| -Line #70- The authors need to remove the word “apparent”. | The word has been removed. | *“….increases within biomarkers denoting satellite cell activity, and patterns in fiber type recruitment (17).”* |
| -Line #73- The authors need to explain why “the large muscle groups of the upper extremity” is a possible limitation for the application of BFRT, especially compared to lower body. | We have edited the sentence for clarity, as suggested. | *“A plausible explanation can be attributed to the anatomical location of the large muscle groups of the upper extremity (e.g., pectoralis major, deltoid, latissimus dorsi) that humpers the proximal application of the cuff and the restriction of the blood flow in contrast to lower extremity where the large muscle groups are located mainly distal (e.g., quadriceps) to the applied occlusive pressure (15).”* |
| Line #75- This last sentence is not a great match for the paragraph. The first two sentences in the paragraph addresses the understudied muscle groups of the upper extremity and provides possible explanations for it. The last sentence, on the other hand, is not related to these. | We thank the reviewer for the comment. The sentence has been moved and rephrased in the following paragraph. | *“Recently emerging evidence in healthy individuals revealed promising outcomes indicating that the use of LL-BFRT may result in increased muscle strength and hypertrophy in muscles located proximal to the BFR cuff application (15, 22). However, the published research until today remains sparse.”* |
| Line #82- Edit as “such as changes in muscle size and strength”. | The sentence has been edited as suggested. | *“….muscle adaptations, such as changes in muscle size and strength, in muscle groups surrounding….”* |
| Methods  -Line #93- The study objective states that “the main objective of this systematic review of randomized controlled trials (RCTs) was to evaluate in healthy individuals the effectiveness of LL-BFRT...”. However, the population for eligibility is different here in line #93. Please reformulate. | We thank the reviewer for picking this up. The sentence in the “Eligibility criteria” section has been edited. | *“1. Population: healthy individuals (no age restriction).”* |
| -Line #136- The authors are suggested to reformulate this sentence. Particularly, the beginning of it (“As measures of intervention effect, given that the outcomes were continuous, ...”) is not very clear. | The sentence has been rephrased for clarity. | *“Using the available outcome measures for muscle size and strength, we calculated standardized mean differences (SMDs) and the associated 95% confidence intervals (95%CI) where data from more than one study was available, and mean differences (MDs) in the case of a single study data availability.”* |
| -Line #152- The authors need to provide references for the categorization of training loads. These subgroups (> 60% 1RM for HL; >40 1RM for LL) do not agree with some of the studies referenced in the Introduction section and are relative. | We thank the reviewer for the comment. Firstly, we apologize for the mistake: LL should be referred as load <40% 1RM. We have corrected the mistake and added refences as requested. | References added in text.  *“…(ii) low intensity exercise (<40% of 1RM).”* |
| -Line #154- The authors may need to explain why they are including studies with a non-exercising control group.  Please also examine the study objective (at the end of the Introduction section) accordingly.  This line also contradicts with the statement in line #191 (“The control group used the same exercises with the BFRT group in every study..”). | We thank the reviewer for the valid comment. Upon reflection, we decided to remove the inclusion of a control group not performing exercise for the present systematic review. The text has been edited to reflect the change. | *Control group not performing exercise was removed for methods, results, Tables and discussion.* |
| Results  -Line #181- In Table 2, third row (Brumitt et al., 2020), please indicate if the significant difference is compared to baseline levels (within-group). | We have edited the main findings in the table to indicate the within- and between-group differences. | *“Significant within-group supraspinatus and external rotators* *strength gains for both BFR and LL-RT group (p<0.001).*  *No between-group differences in supraspinatus (p= 0.750) and external rotator strength (p= 0.708), and supraspinatus tendon thickness (p=0.610)”* |
| -Line #181- In Table 2, 8th row (Yamanak et al., 2012), a comma (,) proceeds the p (p,0.05). Please reformulate. | The typo has been corrected. | *“…for both groups (p<0.05)”* |
| -Line #181- In Table 2, 9th row (Yasuda et al., 2010) “physiological adaptations and main findings” is not clear, please reformulate. | The physiological adaptations and main findings section have been edited for clarity as requested. | *“Significant increase in triceps brachii (8%) and pectoralis major muscle (16%) thickness in favour of the BFRT group (p<0.05) compared to the non-BFRT group*  *Significant increase (p<0.05) in 1-RM bench press strength (6%) in favour of the BFR-T group compared to the non-BFRT group”* |
| -Line #190- Understandably the authors used the term “fatigue”, since the referenced article also used that term. However, it could be more accurate to use a term such as “volitional exhaustion” or “until participants' expressed inability to continue with the task”. | The term has been changed following your recommendation. | *“…participants were instructed to perform the fourth set to volitional exhaustion…”* |
| -Line #223- The referenced study (39) included 40 participants in total and had 4 groups. It could be better if the authors reformulated this sentence similar to this one: “...one study of moderate quality and high risk of bias (39) showed no difference in bench press strength between LL-BFRT (n=10) and no exercise (n=10)...”. | We thank the reviewer for the suggestion. The text has been edited accordingly. | *“Very low certainty evidence from one study of moderate quality and high risk of bias (41) showed no difference in bench press strength between LL-BFRT (n=10) and no exercise (n=10)…”* |
| -Line #259- Add space between “population” and “(40)”. Please also check the whole paper for similar edits (for example line #334). | The space has been added. |  |
| Discussion  -Line #295- The authors summarized the main findings clearly in this paragraph. | We thank the reviewer for the feedback. | *No changes in text.* |
| -Line #312- The authors cited the reference #44 (Centner et al., 2019), however it is not related to the statement made in that sentence. Centner et al., (2019) did not include a LL-RT group, rather they had a HL-RT group. | We thank the reviewer for noticing this mistake. The reference has been replaced. | *Christoph Centner PW, Alber Gollhofer and Daniel König. Effects of Blood Flow Restriction Training on Muscular Strength and Hypertrophy in Older Individuals: A Systematic Review and Meta‑Analysis. Sports Medicine. 2018;49:95–108.* |
| -Line #317- This sentence is not clear. The authors need to explain what they mean by “fatiguing effect of synergistic muscles”. | The text has been edited for clarity. | *“It has been argued that exercising until volitional exhaustion of the prime mover muscles will increase the required activation of the synergistic muscles involved in the performance of an exercise (e.g., the synergistic involvement of the triceps muscle in the performance of a chest press exercise) and that this increase in activation may elicit increases in strength of muscles located proximal to the BFR cuff (15). The authors hypothesized that the increasing fatiguing effect on the triceps muscle under BFR would have caused a greater stress and demand on the pectoralis major muscle to compensate for the loss of force production (15).* |
| -Line #390-422- The authors stated the study limitations and conclusions clearly. The authors may include that the number of studies reviewed did not allow them to perform some of the standard analyses. | We thank the reviewer for the feedback. We have added the limitation a suggested. | *“We also acknowledge that the limited number of the included studies did not allow performance of some of the standard analyses of a systematic review as for example the assessment of publication bias using funnel plots or related regression methods.”* |
| -The authors seem to have cited related and up to date references. | We thank the reviewer for the feedback. | No changes |
| **Reviewer #2: Comments to the Author** | **Authors’ responses** | **Changes in text** |
| Reviewer #2: The manuscript aimed to investigate the effect of blood flow restriction training combined with low load resistance training (LL - BFRT) on upper limb muscle strength and the results of the meta-analysis showed that LL-BFRT had effectiveness in improving muscle strength and size in upper body muscles located proximal to the BFR stimulus in healthy adults. However, the evidence was low certain. Additionally, several issues in this manuscript also should be concerned. | We thank the reviewer for the constructive comments the feedback. | We have made all the requested changes as requested by the reviewer. |
| Major issues：  1. The quality assessment for included papers is very important for “A Systematic Review with meta-analysis”, I suggest “Supplementary Table 2.” should not be supplementary data, it should be put in the manuscript. | Following your recommendation, the table has been moved in the manuscript. | *Table 3 added in the main text.* |
| 2. The authors pooled the papers into a meta-analysis, but the manuscript only showed the effect size of every trial on GRAGE assessment tables. I suggest that forest flots should be used for the evaluation of the total effects and subgroups should be used for different comparisons. | We thank the reviewer for the comment and appreciate the opportunity to clarify and expand our analyses. We agree with the reviewer that simple use of I^2>75% is insufficient to establish heterogeneity. In our analyses we included examination of clinical heterogeneity (demographic and design factors which may plausibly be associated with variation in outcomes) as well as the measures of statistical heterogeneity (<https://training.cochrane.org/handbook/current/chapter-10>) and a leave one out sensitivity analyses. It transpired that the files analysed which had an I^2>75% also failed at least one of these estimates of clinical heterogeneity. We thought it most parsimonious to simply report this in the paper, but we have now expanded this description as requested. We note that all these excluded forest plots are available as supplements.  With regards to your recommendation to pool all studies and then perform a subgroup analysis, we respectfully disagree. Pooling studies assessing strength and muscle volume of the deltoid, the pectoralis major, and latissimus dorsi wouldn’t be appropriate given: a) the observed I^2 values indicating significant heterogeneity, and b) the specificity of the exercises used for training (using BFR or not), the location, and the size of the muscles of interest | *Forest plots are presented only for pooled analyses which did not display excessive heterogeneity. (Heterogeneity was assessed statistically (I^2^<75%), clinically, and using a leave-one-out sensitivity analysis.)* |
| 3. It is hard to read Table 2. In this manuscript, I suggest the characteristics should be: Article, year; country/Region; participants characteristics, sample size (every group); intervention (for every group); duration of intervention; outcomes. | Following your recommendation, the table has been edited accordingly. | *Table 2 updated in text.* |
| 4. I suggest the Methods section should be rewritten as lots of details need to be added, ex. in line 137, “we calculated mean differences (MDs), standardized mean differences (SMDs) where more than one study was available”, it is mean only one study used (MD), more than one study used (SMD)? it’s not correct. | We have expanded the section as requested for the methodology implemented. | *“Using the available outcome measures for muscle size and strength, we calculated standardized mean differences (SMDs) and the associated 95% confidence intervals (95%CI) where data from more than one study was available, after appraising the variability of clinical settings and methods used for strength and muscle size assessment. We calculated and presented mean differences (MDs) in the case of a single study data availability. (Challoumas et al, 2020)”* |
| In lines 139-140, “forest plots were presented if aggregate pooled estimates met the sample and intervention homogeneity criteria.” There was no forest plots in this manuscript except supplementary data, besides, the detail of information on “the sample and intervention homogeneity criteria” should be mentioned. | The relevant forest plots have been added in the manuscript and the used homogeneity criteria have been added for clarity. | *“We pooled pair-wise meta-analyses assessing the same muscle group, using similar methods of strength and muscle volume assessment which evaluated muscle loading of comparable magnitude where recruited participants displayed comparable demographic characteristics, and after leave-one-out sensitivity analyses. However, when heterogeneity was observed in analyses, sensitivity analyses were performed. (Challoumas et al, 2020)”* |
| In lines 147-150, “If considerable between-group statistical heterogeneity……… heterogeneity at baseline for participant characteristics”, we can’t direct move out one article from the meta-analysis pool just because of the high I2, the reason for high heterogeneity should be analyzed, sometimes, subgroups were suggested to be used. | We thank the reviewer for the comment and apologise for the confusion. As pointed out above, we assessed heterogeneity not only statistically, but also clinically. Since none of the sub-group analyses met all heterogeneity criteria, we feel it would be inappropriate to present these pooled analyses (https://training.cochrane.org/handbook/current/chapter-10). | *No changes in text.* |
| 5. in lines 169-170, “of which 179 articles were full text screened. Nine studies met the eligibility criteria and were included.” Two articles were included in the meta-analysis by citation searching but not of which 179 articles. | We thank the reviewer for the comment. The text has been edited accordingly. | *“The search strategy identified 346 potentially relevant publications, of which 23 articles were full text screened.”* |
| 6. Supplementary table 1. only showed the search strategy in Medline, but not other databases. | Search strategy has been added for CINAHL, SportDiscus and Scopus database. | *Additions were made in Supplementary Table.* |
| Minor issues：  1. English writing should be improved. | The language has been checked by a native speaker. |  |
| 2. In the abstract section, "Discussion" should be replaced by "Conclusion", the "LL-HT" group in the “Objective” should be replaced by "LL-RT". | Both corrections were made in text. | *“Conclusion:…..”*  *“(LL-RT, HL-RT)….”* |
| 3. in lines 153-154, “60% 1RM load during exercise (high-load – HL), or (ii) low intensity exercise (>40% of 1RM), or (iii) a control group (no exercise) as a comparator”, the authors should provide the reference. | References have been added as suggested. | *Reference have been added in text.* |
| 4. “Author contributions” should be spilt from the “Acknowledgments” section | The text has been edited. | *Author contribution.* |
