# Supplementary material for: The effects of upper body blood flow restriction training on muscles located proximal to the applied occlusive pressure: A systematic review with meta-analysis
Source: PLoS One. 2023 Mar 23;18(3):e0283309. doi: 10.1371/journal.pone.0283309 (PMC10035935; doi:10.1371/journal.pone.0283309)
Supplement: Attachment — Submitted filename: Reply to Reviewers Comments.docx [file pone.0283309.s005.docx]

| Comment from Reviewer | Reply to Reviewer Comment | Changes to text |
| --- | --- | --- |
| Reviewer #1: The authors have revised the manuscript sufficiently. It may be useful to take a look at the few minor comments below before consideration to publish this research. | We thank the reviewer for the comment | No change |
| -Line #186- One of the studies you included had 6 sessions per week. So, revise as “with 2 to 6 exercise sessions per week”. | We apologise for the typo, text has been revised as recommended | The training duration ranged from 2 to 8 weeks (median=3, IQR 2-3), with 2 to 8 exercise sessions per week (median=2.5, IQR 2-3, range 2 to 6). |
| -Line #187- The median and range values are not correct. Please revise. | We apologise for the typo, text has been revised as recommended | The training duration ranged from 2 to 8 weeks (median=3, IQR 2-3), with 2 to 8 exercise sessions per week (median=2.5, IQR 2-3, range 2 to 6). |
| -Line #310- Better strength and size adaptations compared to which training? | The text has been clarified as requested | The findings of this systematic review suggest that LL-BFRT may result in better strength and size adaptations compared to similar exercise without BFR in muscles proximal to the applied cuff, although the quality of evidence is low, and the findings are mixed |
| Since this is the first paragraph of the Discussion, it is particularly important to give the reader a broad and clear main finding. | We thank the reviewer for the comment. We believe that the first sentence does provide a broad and clear depictions of the main findings of this review. |  |
| -Line #383- Revise the section “by using DEXA” or remove it completely. | The text has been clarified as requested | Eight weeks of rotator cuff training with LL-BFRT may induce greater increases than matched LL-RT in the shoulder region muscle mass and in the whole upper limb where muscle mass is assessed using Dual Energy X-Ray Absorptiometry |
| -Table 2- Brumit et al., (2020) – Participants characteristics: What does “54% trained young adults” mean? | We apologise for the confusion. The text has been corrected | “…53% reported resistance training for the shoulders more than once per week” |
| Reviewer #2: This version of the manuscript has improved so much. | We thank the reviewer for the comment |  |
| The "conclusion" section in the text mentions that caution should be exercised to interpret these findings due to limited evidence. I suggest that the authors mentioned in the abstract before publication. | The text has been corrected as requested | **Conclusion:** With low certainty LL-BFRT appears to be equally effective to HL-RT for improving muscle strength in upper body muscles located proximal to the BFR stimulus in healthy adults. |
